# Invasive pneumococcal diseases in children and adolescents– a single centre experience

**DOI:** 10.1186/1756-0500-7-145

**Published:** 2014-03-13

**Authors:** Christin Schnappauf, Arne Rodloff, Werner Siekmeyer, Wolfgang Hirsch, Ina Sorge, Volker Schuster, Wieland Kiess

**Affiliations:** 1 Department of Woman and Child Health, Hospital for Children and Adolescents, Centre of Paediatric Research, University Hospital of Leipzig, Liebigstraße 20a, D-04103 Leipzig, Germany; 2 Department of Microbiology and Infectious Epidemiology, University of Leipzig, Leipzig, Germany; 3 Department of Paediatric Radiology, University Hospital of Leipzig, Leipzig, Germany

**Keywords:** *Streptococcus pneumoniae*, Invasive pneumococcal disease (IPD), Complications, Children, Serotypes, Pneumococcal vaccination

## Abstract

**Background:**

*S. pneumoniae* is a major cause of meningitis, pneumonia and sepsis in children. In 2006 universal pneumococcal vaccination was recommended in Germany for all children up to their second birthday. We have compared the prevalence and outcome of IPD at a single hospital before and after the introduction of vaccination.

**Findings:**

55 cases of IPD were identified over an 11 year period. Almost half of the patients were younger than 2 years of age. Most of the children were affected by pneumonia. The second highest incidence seen was for meningitis and sepsis. 17 patients exhibited additional complications. Significant pre-existing and predisposing disorders, such as IRAK 4 defect, ALPS or SLE were identified in 4 patients. Complete recovery was seen in 78% of affected children; 11% had a fatal outcome and 11% suffered from long term complications. Only 31% overall had been vaccinated. The most common serotype was 14. Serotypes not covered by any of the current vaccines were also found. Antibiotic treatment commenced with cephalosporins in over 90%.

**Conclusion:**

Frequency of IPD in our hospital did not decrease after initiation of the pneumococcal vaccination. This might be due to vaccinations not being administered satisfactorily as well as to poor education about the need of the vaccination. Pre-existing diseases must be monitored and treated accordingly and rare deficiencies taken into account when IPD takes a foudroyant course. In addition, antibiotic stewardship has been initiated at this hospital centre as a consequence of the high cephalosporin use detected in this study.

## Findings

### Background

Pneumococci are one of the major causes of meningitis, pneumonia and sepsis in children [1-3] and such invasive pneumococcal disease (IPD) is a leading cause of death and significant morbidity in young children in developed countries, particularly those under the age of two [4,5]. The occurrence of distinct serotypes differs with clinical presentation, age and geographical region [6-10]. Since the 1980s a 23-valent polysaccharide vaccine (PPV23) has been available for adults and children older than two years, because of its poor immunogenicity in younger age groups. The pneumococcal conjugate vaccine 7 (PCV 7), which covers the most important seven serotypes causing IPD in children (4, 6B, 9 V, 14, 18C, 19 F, and 23 F) was additionally licensed by the European Union in 2001 [11-14]. In Germany the pneumococcal vaccination has been recommended since July 2006 by the German Standing Vaccination Committee (STIKO) at the Robert Koch Institute for all children up to their second birthday. Three years later, in 2009, PCV 10 which covers three more serotypes (1, 5, 7 F) and PCV 13 for additional coverage of serotypes 3, 6A and 19A were introduced and approved for use in Europe [9,15]. Following the introduction of this vaccination scheme the frequency of IPD with vaccine serotypes has decreased but the number of non-vaccine cases is slowly increasing, suggesting the need for wider serotype coverage in future PCV vaccines [9,11,14,16]. A successful vaccination programme will not only lead to a reduction in the incidence of pneumococcal diseases but will also result in decreased utilisation of antibiotics, thus preserving their efficacy as a primary treatment. It will also slow the development of antibiotic resistance, especially the resistance to penicillin, β-lactams and macrolides, which has steadily become more prevalent worldwide over the last 30 years [1,11,17,18].

Based on the information above and focusing on a single hospital centre (the Hospital for Children and Adolescents, Department of Woman and Child Health, Leipzig, Germany) we have asked:

What is the prevalence of IPD at one hospital centre?

What are the outcomes from the disease?

What co morbidities were present and what disease complications resulted?

Which antibiotic treatments were used?

Which serotypes were prevalent?

How has the vaccination frequency changed since it was generally recommended?

### Materials and methods

#### Case definition

This retrospective study includes all patients up to 18 years of age treated as an inpatient for IPD between 1^st^ January 2001 and 31^st^ December 2011. A diagnosis of IPD was defined as isolation of *S. pneumoniae* from blood, cerebrospinal fluid or pleural puncture cultures, but also by tracheal aspirate which was obtained from some of the paediatric patients requiring tracheal tubes and assisted ventilation.

These definitions corresponded with ICD numbers A40.3 (sepsis due to S. pneumoniae), G00.1 (pneumococcal meningitis) or J13 (pneumonia due to S. pneumoniae).

#### Age

Patients were assigned to one of three age groups to aid further analysis: patients younger than two, patients of two to four years of age, and patients of five to 18 years of age.

#### Acute disease complications and pre-existing conditions

Acute complications of IPD e.g. Waterhouse Friderichsen Syndrome (WFS), haemolytic uremic syndrome (HUS) or disseminated intravascular coagulation (DIC), were routinely assessed for. All incidences of hydrocephalus, seizures, oculomotor palsy and deafness were also assessed. Pre-existing conditions such as unrelated chronic disease, immunodeficiency or other systemic diseases were evaluated.

#### Vaccination

We assessed whether pneumococcal vaccination had taken place and if the immunisation program was completed by the onset of IPD. Since universal pneumococcal vaccination was only routinely recommended in July 2006, the patients were split into two groups for further analysis, those being treated between 2001 and 2006, and those being treated between 2006 and 2011.

#### Serotypes

Serotypes were derived from patient records held at the institute of microbiology, University of Leipzig. Additional serotype information was obtained from Dr Mark van der Linden (National Reference Centre for Streptococci, Department of Medical Microbiology and University Hospital RWTH Aachen).

#### Antibiotic treatment and resistance

Initial, empirical therapy was recorded, as well as any subsequent alteration to antibiotic treatment. Data on antibiotic resistance was also recorded.

#### Outcome

Outcome measures were grouped as follows: complete recovery, significant complications and death.

#### Statistical analysis

Standardised data was recorded in a relational database and descriptive statistics performed utilising SPSS for Windows version 17 (SPSS Inc., Chicago, Illinois, USA).

#### Ethical statement

Ethical approval of this retrospective case study was obtained from the clinical ethical committee of the Children´s hospital of the University of Leipzig and data protection committee approval has been given by the internal review board of the hospital as well.

### Results

#### Demographics

Between 1^st^ January 2001 and 31^st^ December 2011, a total of 55 cases of IPD were detected in children and adolescents aged between 0 and 18 years in a single centre. The frequency of cases between 2001 and 2006 was on average four per year and from 2006 to 2011 five per year. Of the 55 patients diagnosed with IPD, 84% (n = 46) had a positive culture and in 16% (n = 9) the diagnosis was made based on a positive urinary pneumococcal antigen test. In 11% (n = 6) of the cases the diagnosis was based on tracheal secretion cultures (n = 3 in 2005, (n = 2 in 2006, n = 1 in 2011).

The median age was 30 months with a minimum age of one month and a maximum of 17 years. 47% of all cases were younger than two years, 29% between the age of two to four years and 24% older than five years. A total of 76% were therefore younger than five years (n = 18 from 2001 to 2006, n = 24 from 2006 to 2011). 34 patients (62%) were male and 21 patients (38%) female.

#### Disease type

**Figure 1:**
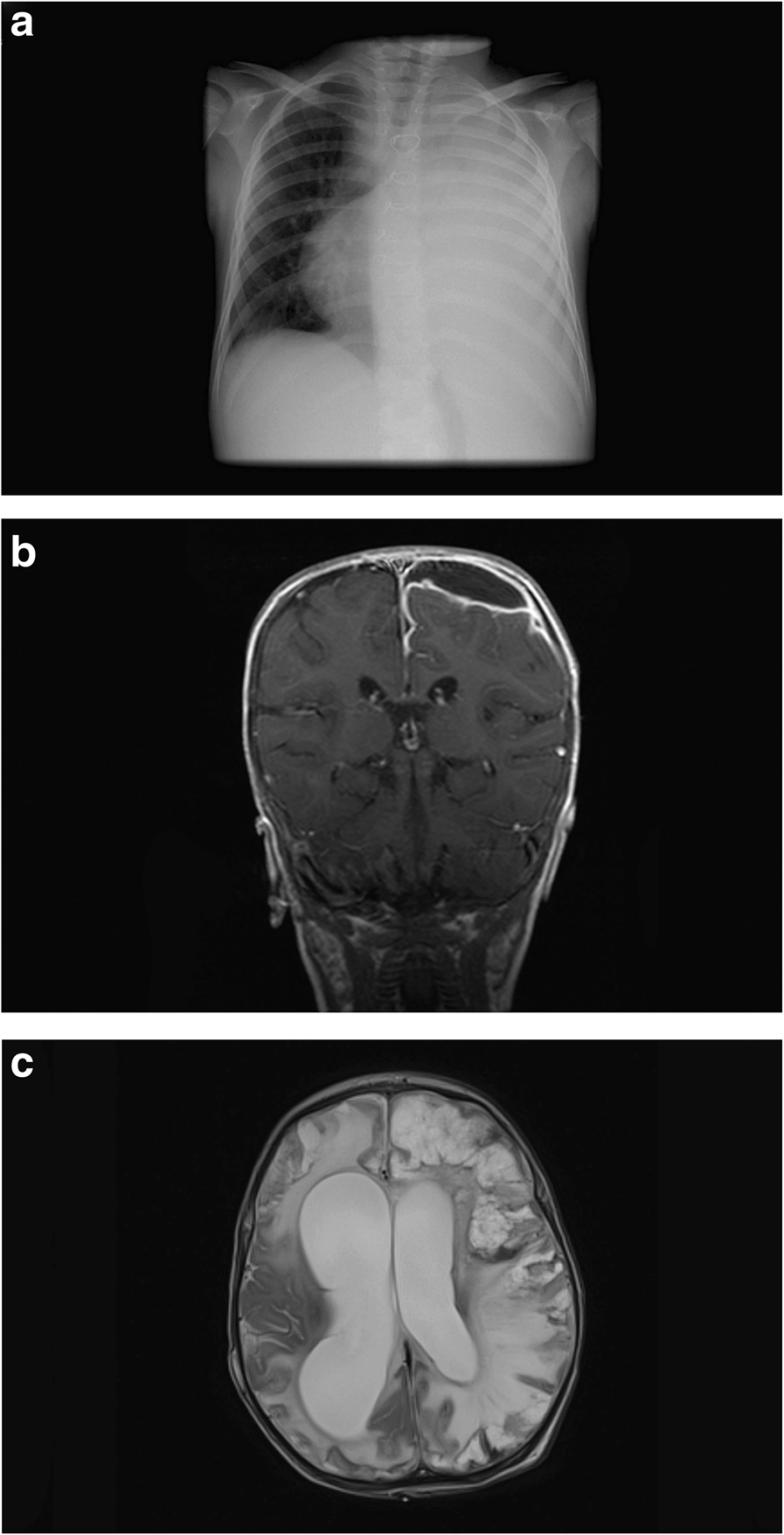
**Radiological findings in three patients with invasive pneumococcal disease. a**: Chest X-ray: Widespread, significant opacification of left hemithorax representing lobar pneumonia with effusion secondary to *S. pneumoniae* in a six year old girl. **b**: Coronary T1-weighted MRI after contrast agent injection: Subdural hygroma in the left hemisphere and increased meningeal enhancement in a one year old boy diagnosed with meningitis secondary to *S. pneumoniae.***c**: T2-weighted MRI without contrast agent: Internal hydrocephalus and pronounced necrosis of the cerebral parenchyma (left > right) in a one month old girl, secondary to IPD with fatal outcome.

**Figure 2:**
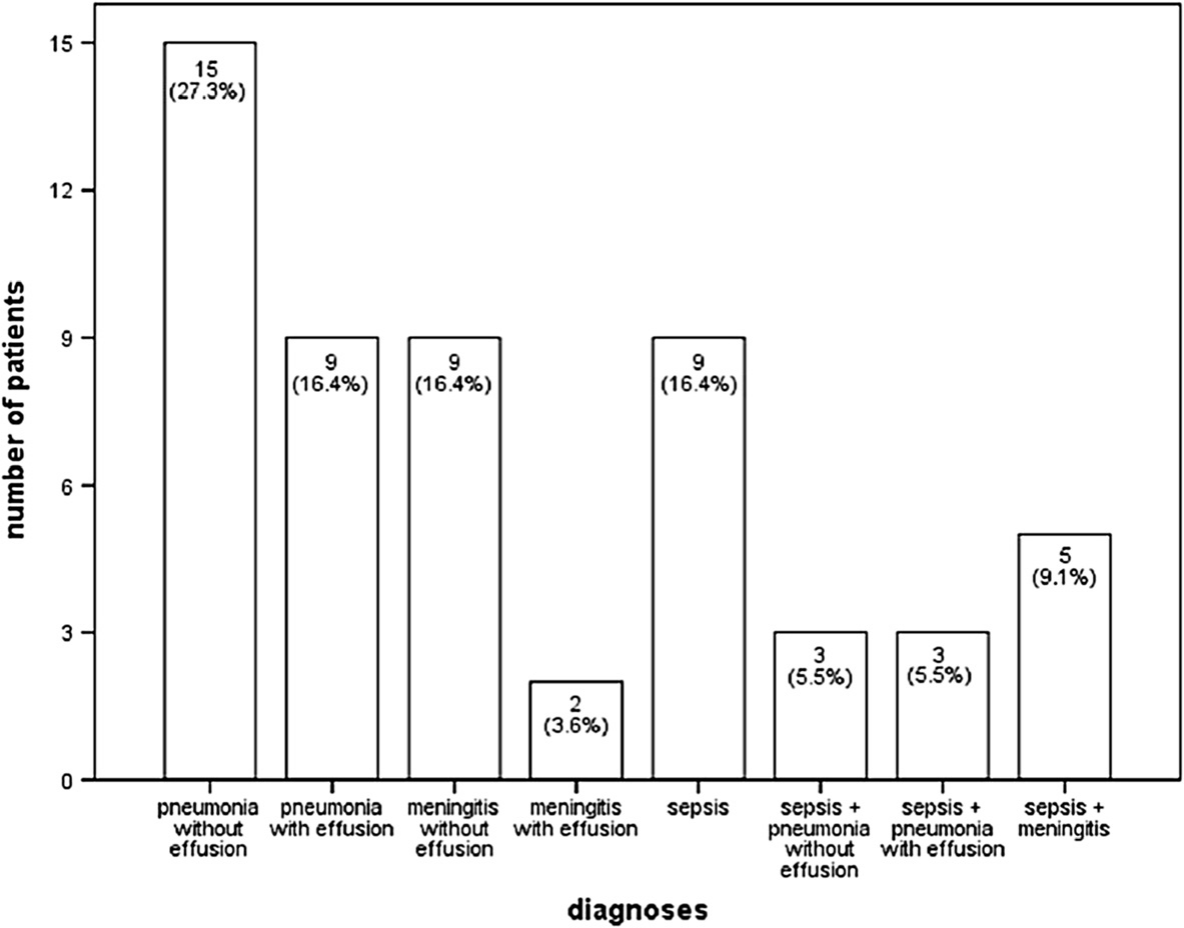
Clinical presentation of invasive pneumococcal disease in 55 children at the Hospital for Children and Adolescents, Department for Women and Child Health, University of Leipzig, Germany from 2001 to 2011.

A total of 24 children (44%) were affected by pneumonia and nine (38%) of those had pneumonia with effusion (Figure 1a). Eleven patients (20%) suffered from purulent meningitis of which two children (18%) had a subdural effusion (Figure 1b). Sepsis due to *S. pneumoniae* was identified in nine cases (16%). Six children (11%) were diagnosed with pneumonia and sepsis at the same time (50% pneumonia with effusion, n = 3) and five patients (9%) with meningitis and sepsis (Figure 2).

#### Complications

Thirteen patients (24%) had acute disease complications. One patient (2%) was diagnosed with haemolytic uremic syndrome (HUS), two patients (4%) with disseminated intravascular coagulation (DIC) and another two children (4%) suffered from HUS and DIC concurrently. Four patients (7%) suffered a seizure and three (6%) a seizure as well as DIC. One oculomotor palsy (2%) and an occlusive hydrocephalus with pronounced necrosis of the cerebral parenchyma (Figure 1c) combined with DIC (2%) were also detected. In the patients evaluated no diagnoses of Waterhouse Friderichson Syndrome (WFS) or deafness were found.

#### Pre-existing disease

In 12 patients significant pre-existing diseases were present. Two siblings were diagnosed with meningitis and sepsis due to *S. pneumoniae*, resulting in similar disease courses with a fatal outcome. The possibility of immunodeficiency was therefore considered and the children were subsequently demonstrated to be homozygous for an interleukin-1 receptor-associated kinase 4 (IRAK 4) deficiency. Another patient was known to have pre-existing autoimmune lymphoproliferative syndrome (ALPS) and went on to make an unremarkable recovery. One further patient had pre-existing systemic lupus erythematosus (SLE) which led to a foudroyant course with fatal outcome.

#### Recovery

Complete recovery was recorded in 43 patients (78%); six children (11%) died and another six (11%) suffered from long-term complications. Most fatalities were found in those under the age of two years (n = 4); whereas only two fatal outcomes were detected in the age group between five and 18 years. The highest numbers of long-term complications were identified within the age group two to four years (n = 3), followed by the < two years (n = 2) and the five to 18 years (n = 1). Three patients suffered from renal failure due to haemolytic uremic syndrome (HUS). One 15 month old child received an allogeneic renal transplantation. One patient with oculomotor palsy secondary to meningitis had a persistent ptosis as well as a gaze paresis. Another child with meningitis and effusion suffered from a right-sided hemiparesis and hyperkinesis, as well as an intention tremor. The relation between pre-existing diseases as well as acute and long-term complications is shown in Table 1.

**Table 1:** Table demonstrating further information on children that developed acute or long-term complications or died (12 out of 55)

**Age (month)**	**IPD**	**Pre-existing disease**	Acute complications*	Long term complication** or death
**1**	Sepsis	None	DIC + hydrocephalus	Death
**7**	Meningitis + sepsis	IRAK-4-deficiency	DIC + convulsive fit	Death
**14**	Meningitis	None	Convulsive fit	Death
**15**	Pneumonia + sepsis	None	HUS	End stage renal disease (ESRD)
**18**	Pneumonia + sepsis	None	HUS	Chronic renal failure
**18**	Meningitis	None	DIC + convulsive fit	Death
**37**	Meningitis with effusion	None	oculomotorius palsy	Ptosis, gaze paresis
**39**	Meningitis	None	None	Right-sided hemiparesis, hyperkinesis + intention tremor
**53**	Pneumonia	None	HUS	Chronic renal failure
**64**	Meningitis + sepsis	IRAK-4-deficiency	DIC + convulsive fit	Death
**79**	Pneumonia with effusion	ALPS	None	None
**156**	Pneumonia + sepsis	SLE	DIC	Death

*Within 2 weeks after onset of disease.

**4 or more weeks after onset of disease.

#### Vaccination and serotypes

In total 17 patients (31%) had previously been vaccinated, 15 (88%) of them between 2006 and 2011 when universal pneumococcal vaccination was generally recommended. Only nine of the 17 vaccinated patients had received the full immunisation of four doses of vaccine before onset of the disease. Despite full vaccination one sibling with IRAK 4 deficiency died. Thirteen children (77%) completely recovered. Three patients (18%) without completed vaccination courses suffered from long-term complications and in these patients we could only identify three serotypes (3, 12 F and 19A). Serotype 12 F is to date not part of any pneumococcal vaccination for children less than two years. Serotypes 3 and 19A are part of PCV 13 but not PCV 7. At the time of diagnosis of those two IPD cases, PCV 7 was the vaccine in general utilisation and therefore did not cover serotype 3 and 19A.

Of 55 cases 21 serotypes were identified; the most frequent one was serotype 14 (n = 5, 9%; Table 2).

**Table 2:** Pneumococcal serotypes and their frequencies in 21 patients with IPD in a single hospital centre

**Serotype**	**3**	**6A**	**6B**	**7 F**	**9 N**	**10A**	**12 F**	**14**	**18C**	**19A**	**19 F**	**23 F**	**34**
**Frequency**	2	1	2	2	1	1	1	5	1	2	1	1	1
**Covered** **by PCV-7**	no	no	yes	no	no	no	yes	yes	no	no	no	yes	no
**Covered** **by PCV-13**	yes	yes	yes	yes	no	yes	yes	yes	yes	yes	yes	yes	no
**Vaccinated**	n = 1	n = 0	n = 0	n = 0	n = 0	n = 0	n = 1	n = 0	n = 0	n = 2	n = 0	n = 0	n = 0

#### Antibiotic therapy

In 51 cases (93%) initial antimicrobial therapy was performed with cephalosporins (cefuroxime n = 30, 59%; cefotaxime n = 18, 35%; ceftazidime n = 3, 6%). In the remaining cases initial antibiotic treatment (each n = 1, 2%) was carbapenems or glycopeptides (vancomycin) and in two patients (4%) aminopenicillins (ampicillin). In 15 cases (27%) a change of therapy was deemed necessary based on culture results or microbiological advice, often leading to combined antibiotic regimen (n = 5, 9%) or a switch to carbapenems (n = 7, 13%). In two patients treatment was changed to a cephalosporin of a higher group and in another patient to a lincosamide antibiotic. Microbiological findings revealed antibiotic resistance in 10 patients (18%), half of which demonstrated resistance to polymyxins and in two cases (4%) to macrolides. The rate of resistance to lincosamides, ketolides, aminoglycosides, aminopenicillins and second generation cephalosporins was less than 3%. Intermediate resistance was identified in 11 cases (20%); six patients (11%) for second-generation fluoroquinolones, four patients (7%) to macrolides and one (2%) to penicillin.

### Discussion and conclusion

The incidence of IPD in this single hospital centre has not significantly changed since the introduction of the general vaccination programme in 2006. It seems that more IPD cases were diagnosed between 2006 and 2011 than in the pre-vaccination era between 2001 and 2006, with a maximum of 11 cases in 2011. Even though studies overall show that IPD has decreased [4,16] why have the diagnoses of IPD increased in this single centre? Looking critically into possible reasons, one theory to explain the high number of IPDs detected between 2006 and 2011 could be the use of new diagnostic methods after 2006 such as the urinary antigen test. Nine of the 11 cases in 2011 were found to be diagnosed with this test rather than by culturing of *S.pneumomiae*. In contrast to this theory, this test has a high sensitivity but quite poor specificity, potentially resulting in false-positive results which may drive up the number of diagnoses and distort the true number of IPD detections [19,20]. Increased attention to the disease and a subsequent higher rate of blood-cultures could also lead to this apparent increase in diagnoses [16,21]. Furthermore, another important reason could be the low frequency of vaccinations: only 17 (33%) of the total 55 patients had been vaccinated. In most European countries pneumococcal vaccination with PCV7 was recommended since 2006. Most of the countries report a decrease of vaccine serotypes but higher numbers of non-vaccine serotypes were also noticed [22]. The serotypes (6B, 14, 18C, 23 F) identified in nine of the children in the pre-vaccination era could have been mainly covered by PCV 7 which underlines the need of the introduction of pneumococcal vaccination in that time. A similar situation was seen between 2006 and 2011. In six cases the identified serotypes (3, 6A, 7 F, 19A) would have been covered by PCV 13 but the children were suffering from IPD before 2009. This was therefore before the introduction of PCV 13 which might have prevented the disease. On the other hand in four patients during 2001 and 2011 the serotypes 10A, 12 F, 34 and 9 N were identified which are to date not part of any pneumococcal vaccination for children under two years of age.

Serotype 14 is one of the most common serotypes in Europe in children under 18 years of age [4,8,23,24] and was also present in five patients in our study. Kaplan et al. state that serotype 19A is currently the most common serotype in the United States after PCV7 introduction [25]. In Germany a higher incidence of IPD caused by serotype 19A was also noticed in 2008/2009 whereas 19A isolates remained below 5% before 2006. The cause for this might be due to increased reporting after introduction of PCV7, but an increased use of cephalosporins is also a possibility for the change in 19A epidemiology [22]. In our hospital two cases were identified in 2008. Both children were vaccinated with PCV7 which does not cover serotype 19A. Overall 93% of the children were treated with cephalosporins. Data regarding antibiotic use in Germany show a significant increase in cephalosporin use which may have led to an increase of serotype 19A [22].

Pre-existing diseases especially immunodeficiencies and autoimmune diseases may increase the risk of IPD. The ALPS found in one of the children usually develops in early childhood and is an autoimmune condition caused by an inherited defect in the immune system secondary to an error in the Fas or FasL gene. The Fas gene normally functions to induce lymphocyte apoptosis and such defects reduce cell death among lymphocytes after infection, leading to a higher number of lymphocytes that continue to create antibodies [26]. Patients with ALPS should receive all childhood vaccinations including pneumococcal vaccination to reduce the number of potential infections. The child in this study did not receive pneumococcal vaccination due to suffering from IPD one year before the vaccination in Germany was generally recommended. In this instance ALPS was only diagnosed when the child was already suffering from IPD and therefore no preventative, early immunisations had been administered.

One patient was known to have SLE, which can increase the risk and frequency of S*. pneumoniae* infections due to significantly reduced opsonisation of *S. pneumoniae* with C3b/iC3b in serum [27-29]. Besides SLE itself being a systemic autoimmune disease which can affect any part of the body, it leads to multisystem organ failure and death [27-29] as it was also shown in our patient who died of pneumococcal pneumonia and sepsis after 6 days of hospital treatment at the age of 13 years.

Two siblings were affected by IRAK-4 deficiency, a rare primary immunodeficiency with severe impairment of Toll-like receptor (TLR) and interleukin-1 receptor-mediated immunity [30-33]. Patients with IRAK-4 deficiency are highly susceptible to severe and often fatal IPD. In many IRAK-4-deficient patients clinical and laboratory signs of inflammation (such as CRP, IL-6, leukocytosis) develop slowly even in instances of severe infection, as seen in the first sibling in our study [31-33]. In contrast, the older sibling with IRAK-4 deficiency demonstrated raised levels of CRP (192 mg/l; normal < 5 mg/l) and IL-6 (1983 pg/ml; normal < 7 pg/ml), potentially indicating a prolonged infection, a disease course which has also been reported in other IRAK-4-deficient patients with severe IPD [31].

The IRAK 4 deficient patients and the patient with SLE comprise half the fatalities in this study, potentially confirming that immunodeficiency and autoimmune co-morbidities influence the outcome from IPD. The other three fatalities were due to serotypes that are covered by PCV 7 or PCV 13 and therefore could have been prevented.

In three patients renal failure caused by HUS was diagnosed. *S. pneumoniae* associated HUS (SP-HUS) is a serious complication of IPD associated most frequently with pneumonia and empyema due to serotypes not included in PCV-7, particularly serotype 3 [34]. Subsequently, SP- HUS rates vary among different countries across the world, depending upon vaccine type administered [34-37]. In our study three cases of HUS occurred after pneumococcal vaccination had generally been recommended.

During our study period from 2001 to 2011 76% of the patients were younger than five years of age. Splitting this time period into pre and post vaccination era, there is not much difference to recognise. From 2001 to 2006 18 children were found to be younger than five and from 2006 to 2011 24 children suffered from IPD. Most of those patients were younger than two years, confirming that children under the age of two are the most susceptible group for the development of IPD [1,4,12]. Further, 67% of all fatalities and 33% of long term complications occurred in this age group, emphasizing the importance of pneumococcal vaccination and the necessity for improving vaccination rates in children under two years of age in our local area.

Even though the case numbers in our study are too small to show representative statistics, it underlines the clinical relevance of IPD with a significant mortality of 11%. The analysis also shows the importance of pre-existing diseases influencing the outcome and the importance of considering rare deficiencies like the IRAK 4 defect, SLE or ALPS. An important limitation of our study was also the small numbers of identified serotypes due to IPD reporting not being mandatory in Germany at that time. However, information about serotypes that could be analysed, underline the importance of the recommended pneumococcal vaccination to prevent long term complications or death.

Overall we hypothesize that current vaccination protocols in our single centre have either not been implemented widely enough or that the efficacy of such programmes are not good enough to date, to successfully prevent IPD. In addition, based on the detection of a high rate of cephalosporin use as a primary treatment line in IPD in this hospital centre, antibiotic stewardship has been initiated formally in our hospital.

## Abbreviations

ALPS III: Autoimmune lymphoproliferative syndrome type III; CRP: C-reactive protein; DIC: Disseminated intravascular coagulation; ESRD: End stage renal disease; HUS: Haemolytic uremic syndrome; IPD: Invasive pneumococcal disease; IRAK 4: Interleukin 1-receptor associated kinase 4; PCV: Pneumococcal conjugate vaccine; SLE: Systemic lupus erythematosus; SP-HUS: Streptococcus pneumoniae - haemolytic uremic syndrome; STIKO: Ständige Impfkommission (German Standing Vaccination Committee); TLR: Toll-like-receptor; WFS: Waterhouse Friderichsen Syndrome.

## Competing interests

All authors report no competing interests to declare.

## Authors’ contributions

CS collected the data, performed the statistical analysis and drafted the manuscript. AR provided serotype data. WH and IS helped choosing relevant images and did the reports. VS and WK conceived of the study, and participated in its design and coordination and helped to draft the manuscript. All authors read and approved the final manuscript.
